# Ectopie rénale croisée de découverte fortuite chez l'adulte: à propos d'un cas

**DOI:** 10.11604/pamj.2019.33.178.11152

**Published:** 2019-07-08

**Authors:** Anass Elbouti, Mostafa Rafai, Said Jidane, Ahmed Belkouch, Hicham Bakkali, Lahcen Belyamani

**Affiliations:** 1 Pôle des Urgences Médico-chirurgicales, Hôpital Militaire d'Instruction Médicale V, Faculté de Médecine et de Pharmacie de Rabat, Université Mohammed V, Rabat, Maroc

**Keywords:** Ectopie rénale croisée, cholécystite, découverte fortuite, Crossed renal ectopia, cholecystitis, fortuitous discovery

## Abstract

L'ectopie rénale croisée est une anomalie congénitale rare, dans laquelle, les deux reins se situent du même coté avec un des uretères, de longueur adaptée au siège du rein, qui croise la ligne médiane pour s'implanter dans la vessie du coté opposé. L'existence d'une fusion rénale entre les deux parenchymes est fréquente, et cette anomalie est le plus souvent asymptomatique et de découverte fortuite. Nous rapportons l'observation d'un patient de 36 ans sans antécédents (ATCD) pathologiques notables, présentant une ectopie rénale croisée découverte fortuitement suite à une cholécystite aigue compliquée d'abcès péri vésiculaire. Sur la base de cette observation et des données de la littérature, nous discuterons les aspects étiopathogéniques, cliniques, radiologiques et thérapeutiques de cette malformation.

## Abstract (en)

Crossed renal ectopia is a rare congenital abnormality, in which the two kidneys are located in the same side. One of the ureters, whose length is adapted to the kidney seat, crosses the median line to implant in the bladder on the opposite side. Renal fusion of the two parenchymas is common and this abnormality is most often asymptomatic and fortuitously discovered. We report the case of a 36-year old patient with no previous pathological history, presenting with crossed renal ectopia incidentally discovered after acute cholecystitis complicated by perivescicular abscess. On the basis of this observation and of the data from the literature, we here discuss the etiopathogenic, clinical, radiological and therapeutic features of this malformation.

## Introduction

L'ectopie rénale croisée est une anomalie congénitale rare, elle a été décrite par WILMER en 1938 [1], implique qu'un des deux reins siège du côté controlatéral, l'uretère du rein ectopique croise la ligne médiane pour s'aboucher dans la vessie du coté opposé, l'existence d'une fusion parenchymateuse est fréquente (85 à 90%). Cette anomalie est le plus souvent asymptomatique et de découverte fortuite. Nous rapportons l'observation d'un patient de 36 ans porteur d'une ectopie rénale croisée découverte fortuitement suite à une cholécystite aigue.

## Patient et observation

**Figure 1:**
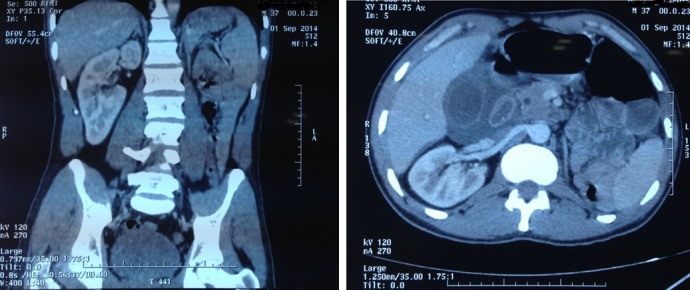
Ectopie rénale croisée avec fusion parenchymateuse des deux reins

Il s'agissait d'un patient de 36 ans sans antécédents pathologiques particuliers, s'est présenté au service des urgences pour des douleurs de l'hypochondre droit et de l'épigastre d'apparition brutale à type de coliques avec une irradiation vers la fosse iliaque droite et en hémi-ceinture associée à des nausées, vomissements et brulures mictionnelles dans un contexte fébrile. L'examen clinique trouvait un patient conscient, tachycarde à 100 battements/min, normo tendue, eupnéique, fébrile à 38,5°C. La palpation abdominale notait une douleur de l'hypochondre droit qui bloque l'inspiration profonde (signe de Murphy positif) avec une défense localisée au niveau de l'hypochondre droit et l'épigastre. Le bilan biologique réalisé aux urgences retrouve une hyper-leucocytose à 21000/mm^3^, cytolyse modérée prédominante sur l'ALAT avec élévation de la CRP (36 mg/l). Le reste du bilan comprenant ionogramme, lipasémie et ECBU est normal. L'échographie abdominale montrait une vésicule biliaire augmentée de taille à paroi épaissie avec épanchement péri vésiculaire et Murphy échographique positif. La loge rénale gauche est vide et le rein droit augmenté de volume avec individualisation de 02 systèmes pyéliques. Le scanner abdominale a été demandé et a objectivé une cholécystite aigue avec infiltration de la graisse adjacente et fine lame d'épanchement. Par ailleurs le scanner abdominale a permis de découvrir une ectopie rénale croisée avec fusion parenchymateuse des deux reins et individualisation de l'abouchement des deux uretères après reconstruction a l'aide de l'algorithme MIP Figure 1. Une cholécystectomie a été réalisée en urgence, les suites opératoires étaient simples, et le patient adressé ensuite au service d'urologie pour complément de prise en charge.

## Discussion

L'ectopie rénale croisée est une malformation congénitale rare, est due à une anomalie dans le développement embryonnaire du bourgeon urétéral et du blastème métanephrique entre la quatrième et la huitième semaine de gestation. Son incidence réelle n'est pas connue car le plus souvent asymptomatique, sa prévalence a été estimée sur des séries autopsiques entre 1/1300 et 1/7600, se caractérise par une prédominance masculine avec un sexe ratio de 3hommes/femme [2]. Sur le plan clinique, cette malformation peut demeurer asymptomatique et être découverte fortuitement à la suite de la réalisation d'explorations radiologiques pratiquées pour un autre motif; comme c'est le cas dans notre observation où le diagnostic a été porté dans un contexte de cholécystite aigue. Dans ce cas, les deux reins sont souvent indemnes, par ailleurs, l'ectopie rénale croisée peut se révéler par des douleurs abdominales atypiques de siège variables, parfois associés a des troubles gastro-intestinaux à type de ballonnement, nausées et constipation; des troubles urinaires à type d'hématurie, pyélonéphrites a répétition ou la découverte d'une masse palpable peuvent attirer l'attention sur une affection urologique et inciter à pratiquer les investigations qui conduiront au diagnostic de rein ectopique [2,3]. Il est donc nécessaire de pratiquer un examen échographique abdominal devant tous les syndromes abdominaux atypiques [3]. L'examen diagnostique de choix de l'ectopie rénale croisée est l'échographie abdominale. En faite l'échographie abdominale est un examen simple et non invasif, reproductible et à cout peu élevé mérite d'être pratiquée en première intention devant une suspicion d'ectopie rénale; et permet de poser le diagnostic d'un rein ectopique avec appréciation de la qualité et la valeur du rein par l'étude de l'index cortical et surtout une étude précise des rapports du rein ectopique avec les organes de voisinage [4]. La tomodensitométrie abdominale permet de confirmer le diagnostic, l'existence d'une symphyse et l'individualisation de l'abouchement des deux uretères après reconstruction à l'aide de l'algorithme MIP. L'UIV elle fournit des renseignements souvent suffisants et décisifs, elle affirme le diagnostic en montrant la vacuité d'une fosse lombaire et laissant apparaitre l'imprégnation du rein ectopique; elle détermine le coté, le siège de la malposition et précise la valeur fonctionnelle du rein. L'artériographie recommandée si une intervention chirurgicale apparait nécessaire, permet de prévoir les difficultés que l'on rencontrera au cours de l'intervention. Sur le plan thérapeutique, la découverte d'une ectopie rénale croisée asymptomatique n'implique pas obligatoirement des complications ultérieures, l'abstention opératoire doit être de mise sous couvert d'une surveillance échographique et d'une analyse périodique des urines, les reins n'ont pas besoin d'être séparés [2,5].

## Conclusion

L'ectopie rénale croisée est généralement asymptomatique, ou présente des signes cliniques peu spécifiques, l'échographie est l'examen diagnostique de choix, l'abstention opératoire doit être de mise en absence de complications, et la surveillance s'appuie sur un suivi échographique périodique.

## Conflits d’intérêts

Les auteurs ne déclarent aucun conflit d'intérêts.
